# Automated phosphopeptide enrichment from minute quantities of frozen malignant melanoma tissue

**DOI:** 10.1371/journal.pone.0208562

**Published:** 2018-12-10

**Authors:** Jimmy Rodriguez Murillo, Magdalena Kuras, Melinda Rezeli, Tasso Milliotis, Lazaro Betancourt, Gyorgy Marko-Varga

**Affiliations:** 1 Clinical Protein Science & Imaging, Department of Clinical Sciences (Lund), Department of Biomedical Engineering, Lund University, Lund, Sweden; 2 Translational Science, Cardiovascular Renal and Metabolism, IMED Biotech Unit, AstraZeneca, Gothenburg, Sweden; NIH, UNITED STATES

## Abstract

To acquire a deeper understanding of malignant melanoma (MM), it is essential to study the proteome of patient tissues. In particular, phosphoproteomics of MM has become of significant importance because of the central role that phosphorylation plays in the development of MM. Investigating clinical samples, however, is an extremely challenging task as there is usually only very limited quantities of material available to perform targeted enrichment approaches. Here, an automated phosphopeptide enrichment protocol using the AssayMap Bravo platform was applied to MM tissues and assessed for performance. The strategy proved to be highly-sensitive, less prone to variability, less laborious than existing techniques and adequate for starting quantities at the microgram level. An Fe(III)-NTA-IMAC-based enrichment workflow was applied to a dilution series of MM tissue lysates. The workflow was efficient in terms of sensitivity, reproducibility and phosphosite localization; and from only 12.5 μg of sample, more than 1,000 phosphopeptides were identified. In addition, from 60 μg of protein material the number of identified phosphoproteins from individual MM samples was comparable to previous reports that used extensive fractionation methods. Our data set included key pathways that are involved in MM progression; such as MAPK, melanocyte development and integrin signaling. Moreover, tissue-specific immunological proteins were identified, that have not been previously observed in the proteome of MM-derived cell lines. In conclusion, this workflow is suitable to study large cohorts of clinical samples that demand automatic and careful handling.

## Introduction

Malignant melanoma (MM) is a type of cancer that has a high rate of incidence in many countries [1]. Along with an unfavorable prognosis, MM lacks any well-established biomarkers to aid in early detection, progression and treatment [1,2]. In this context, the use of -*omic* approaches is essential to further understand the disease. In particular, proteomics has led to the identification of disease-related mechanisms by exploring the proteomes from melanoma-derived cell lines, blood and patient tumors [3]. To deeply cover several aspects of MM including the discovery of biomarkers of clinical relevance, current clinical proteomic studies primarily consider large MM patient cohorts where samples have been correctly collected, stored and documented [4].

The results acquired from cell line-based studies do not necessarily reflect the complexity of the disease. Exploring MM in patient tumors is crucial because the heterogeneity of the tissue and the microenvironment of the surrounding cells is taken into consideration [5–7]. In addition, the analysis of individual patients could be integrated with clinical data to acquire more reliable information on biomarker candidates [4].

Phosphorylation is highly-relevant in many biological processes (signaling pathways, metabolism, cell growth, *etc*.) [8] and in particular, is central to the role of the RAS/RAF/MAPK pathway in MM proliferation and survival [3]. Thus, proteomics of MM has focused on the analysis of the phosphoproteome. In most cases, these studies have been performed using cell lines because of ease of handling, replication power and large quantities of available protein to perform proteomic experiments [9–11]. The main phosphoproteomics strategies have taken advantage of these characteristics and have been shown to be reliable in detecting and identifying thousands of phosphoproteins in a single experiment [12].

Amongst the most-common methodologies used to enrich phosphopeptides from samples are IMAC [13] and titanium dioxide (TiO_2_) [14], and combinations thereof with fractionation techniques such as strong-cation exchange (SCX) [15], hydrophilic interaction liquid chromatography (HILIC) [16] or basic reversed-phase chromatography [17]. Although these approaches have been successful, some limitations should be considered. These include: reproducibility, the large quantities of starting material required, very labor-intensive manual steps in such a complex experiment; and the lack of automation.

Such limitations are particularly challenging when investigating MM-derived patient tissue. As such, the enrichment protocols that are often utilized are susceptible to sample variability and material loss. In this context, the Agilent AssayMAP Bravo platform has been reported to have the ability to conduct protein digestion, peptide desalting and phosphopeptide enrichment as an automated workflow [18–21]. This automation enables the simultaneous processing of multiple samples and thereby increases both efficiency and reproducibility. In particular, the Fe(III)-IMAC-based phosphorylation enrichment protocol has shown outstanding performance in detecting thousands of phosphopeptides from only a few micrograms of protein sample with high selectivity [19,21]. This technology thus represents an alternative means to study MM tissues.

Our group recently reported a study on protein extraction, digestion, peptide enrichment and fractionation of MM tumor samples using the Agilent AssayMap Bravo platform (submitted manuscript). In the present work, the overall performance of this instrument for the phosphoproteomic analysis of MM lymph node tumors deposited in a Biobank was evaluated. Automated phosphopeptide enrichment on the Bravo AssayMap platform revealed a very high selectivity (85–98%) for all the input material that was assessed. Additionally, reproducible and consistent results were obtained as shown by the high correlation between experimental replicates. More importantly, the high sensitivity achieved with this technology led to the generation of results that were comparable to studies where at least 12-fold larger quantities of starting material from melanoma cultured cells were used. Finally, specific biological pathways that have not been previously described in melanoma cultured cells were identified in the MM-derived tissue.

## Materials and methods

This study was approved by the Regional Ethical Committee at Lund University, Southern Sweden, approval numbers: DNR 191/2007, 101/2013 and 2015/266, 2015/618. All patients within the study provided written informed consent. The MM tumor tissues used had been deposited in the Biobank located at the Department of Biomedical Engineering (Lund University, Sweden). Samples corresponded to lymph node metastases from melanoma patients undergoing surgery at Lund University Hospital, Sweden.

### Protein extraction, digestion and automated C18 desalting workflow

Protein extraction was performed on sectioned, fresh-frozen tissues from human MM lymph node metastases tissues (10 μm) using the Bioruptor plus, model UCD-300 (Dieagenode). A total of 20 tissue sections were lysed in 100 μL lysis buffer containing 4 M urea and 100 mM ammonium bicarbonate. After briefly vortexing, samples were sonicated in the Bioruptor for 40 cycles. Each cycle consisted of 15 s at high power and 15 s without sonication at 4°C. The samples were then centrifuged at 10,000 ×*g* for 10 min at 4°C. According to the instructions provided by the manufacturer, the protein content in the supernatant was determined using the colorimetric micro BCA Protein Assay kit (Thermo Fisher Scientific, Rockford, IL).

Proteins were reduced with 10 mM DTT for 1 h at room temperature (RT) and sequentially alkylated with 20 mM iodoacetamide for 30 min in the dark at RT. To decrease the urea concentration, the samples were then diluted approximately seven times with 100 mM ammonium bicarbonate. Digestion was performed in two steps. Proteins were firstly incubated with Lys-C at a 1:50 (w/w) ratio (enzyme:protein) for 5 h and then trypsin was then added at a 1:50 (w/w) ratio and the mixture incubated overnight at RT. The reaction was quenched by adding 20% TFA to a final concentration of ~1%. Peptides were desalted on the AssayMAP Bravo (Agilent Technologies) platform using the peptide cleanup v2.0 protocol. Briefly, C18 cartridges (Agilent, 5 μL bed volume) were primed with 100 μL 90% acetonitrile (ACN) and equilibrated with 70 μL 0.1% TFA at a flow rate of 10 μL/min. The samples were loaded at 5 μL/min, followed by an internal cartridge wash with 0.1% TFA at a flow rate of 10 μL/min. Peptides were eluted with 30 μL 80% ACN, 0.1% TFA and diluted to 200 μL with the same buffer for subsequent phosphopeptide enrichment.

### Automated Fe(III)-IMAC-based workflow

Following the Phospho Enrichment v2.0 protocol, phosphorylated peptides were enriched using 5 μL Fe(III)-NTA cartridges on the AssayMAP Bravo platform. The cartridges were primed with 100 μL 50% ACN, 0.1% TFA at a flow rate of 300 μL/min and equilibrated with 50 μL loading buffer (80% ACN, 0.1% TFA) at 10 μL/min. Samples eluted from the C18 material were loaded onto the cartridge at 3.5 μL/min. The columns were washed with 50 μL loading buffer and the phosphorylated peptides were eluted with 25 μL 5% ammonia directly into 10 μL 50% formic acid. Samples were lyophilized in a vacuum concentrator and stored at -80°C until analysis by LC-MS/MS.

### Automated off line high pH fractionation

Reversed-phase high pH (HpH) fractionation of the MM digests were performed on the Bravo AssayMAP system using the Fractionation v1.0 protocol. The pH of the digested samples were adjusted to 10 with 200 mM ammonium formate. RPS cartridges (Agilent, 5 μL bed volume) were primed with 100 μL 90% ACN and equilibrated with 70 μL 20 mM ammonium formate (pH 10) at a flow rate of 10 μL/min. Samples were loaded at 5 μL/min followed by an internal cartridge wash with 20 mM ammonium formate at 10 μL/min. Elution was performed in six steps with increasing concentrations of ACN in ammonium formate: 4% (F1), 10% (F2), 15% (F3), 20% (F4), 25% (F5) and 80% (F6); 30 μL was eluted into 50 μL of pre-existing buffer containing 50% ACN, 0.1% TFA. The flow through (FT) was collected, processed on C18 cartridges and analyzed as an additional fraction.

### LC-MS/MS analysis

LC-MS/MS analysis was performed on a Thermo Easy-nLC 1000 HPLC coupled on-line to a Q Exactive mass spectrometer (Thermo Scientific, San Jose, CA) or on an Ultimate 3000 HPLC coupled to a Q Exactive HF-X mass spectrometer (Thermo Scientific, San Jose, CA). Peptides were loaded onto a trap column (Acclaim1 PepMap 100 pre-column, 75 μm, 2 cm, C18, 3 mm, 100 Å, Thermo Scientific, San José, CA) and then separated on an analytical column (EASY-Spray column, 25 cm, 75 μm i.d., PepMap RSLC C18, 2 mm, 100Å, Thermo Scientific, San José, CA) using a 120 min ACN gradient in 0.1% formic acid at a flow rate of 300 nL/min and a column temperature of 45°C. Settings on the Q Exactive mass spectrometer were as follows: data-dependent analysis (DDA) selection of the 10 most intense ions for fragmentation, full MS scans at *m/z* 375–1,750 with a resolution of 60,000 at *m/z* 200, target AGC value of 3×10^6^ and an injection time (IT) of 60 ms, fragmentation in the HCD collision cell with an NCE of 25 and MS/MS spectra acquisition in the Orbitrap analyzer at a resolution of 35,000 (at *m/z* 200) with a maximum IT of 120 ms and dynamic exclusion of 30 s. Settings on the Q Exactive HF-X mass spectrometer were as follows: DDA selection of the 15 most intense ions for fragmentation, full MS scans at *m/z* 375–1,750 with a resolution of 120,000 at *m/z* 200, a target AGC value of 3×10^6^ and IT of 100 ms, fragmentation in HCD collision cell with an NCE of 25 and MS/MS spectra acquisition in the Orbitrap analyzer at a resolution of 60,000 (at *m/z* 200) with a maximum IT of 120 ms and dynamic exclusion of 30 s.

### Data analysis

Raw files were processed with Proteome Discoverer 2.1 (Thermo Scientific). The search was performed against the *Homo sapiens* UniProt revised database with the Sequest HT search engine. Cysteine carbamidomethylation was set as fixed modification and methionine oxidation, protein N-terminal acetylation and phosphorylation of serine, threonine and tyrosine were set as variable modifications; peptide mass tolerance for the precursor ions and MS/MS spectra were 10 ppm and 0.02 Da, respectively. A maximum of two missed cleavage sites were accepted. The ptmRS algorithm was used to score phosphorylation sites with a site probability threshold >75. Filtering was performed at a 1% false discovery rate (FDR) for both peptides and proteins. For statistical analyses, label-free quantification (LFQ) data was log_2_-transformed and normalized to column median as standard methods. Analyses such as Pearson correlation, ANOVA and clustering were performed using the Perseus software.

## Results and discussion

**Fig 1:**
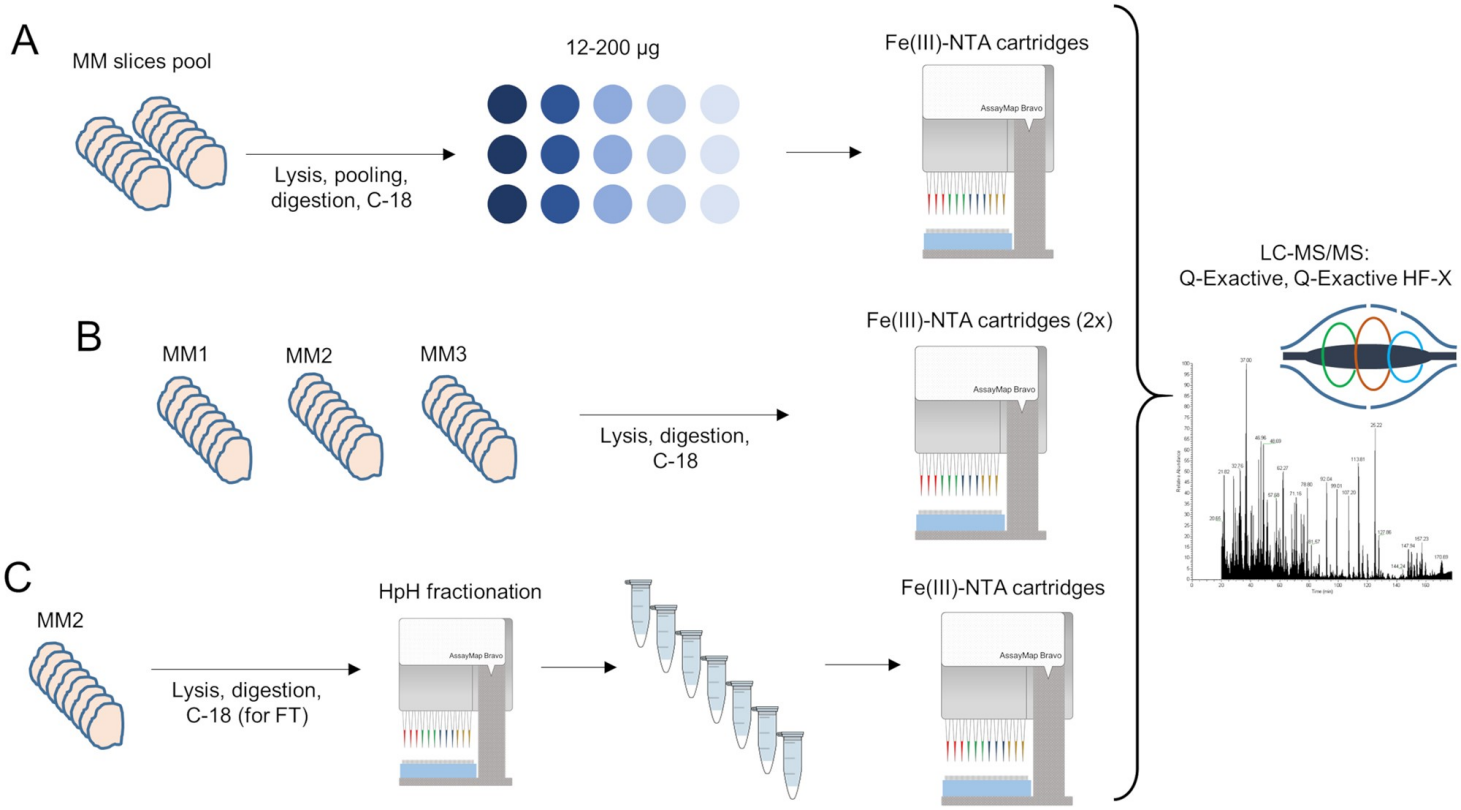
Overview of the workflows performed for the phosphoproteomic analyses. (A) Phosphoproteome analysis with varying quantities of MM peptides extracted from pooled tissues; each enrichment was acquired in technical triplicates. (B) Phosphopeptide enrichment of digested MM tissues performed on three different tissues in biological duplicates. (C) Phosphopeptide enrichment combined with HpH fractionation for a single MM sample (one biological replicate).

Experiments were conducted on proteins obtained from the lysis of fresh, frozen MM tissues (20 sections per tissue). Three experiments were performed: (A) sensitivity assessment of automated enrichment from pooled MM lysates; (B) analysis of the MM phosphoproteome from individual tumor samples; and (C) deep coverage of the MM phosphoproteome using HpH fractionation (Fig 1).

### Sensitivity analysis of automated phosphopeptide enrichment

**Fig 2:**
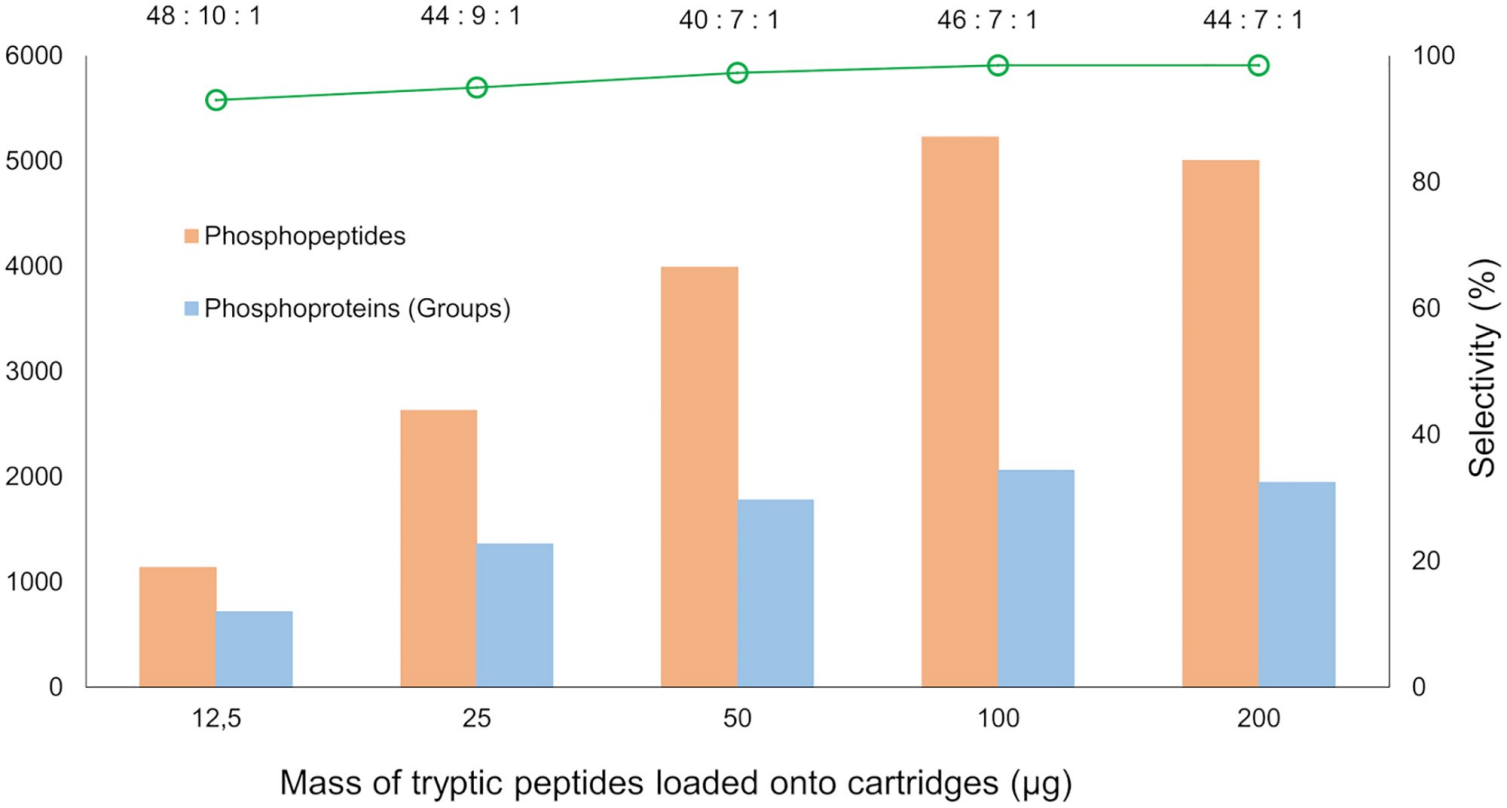
Sensitivity curve of the automated phosphoproteome analysis. Phosphopeptide enrichment was performed with a pooled MM digest using 12.5 to 200 ug of input material. Values expressed at the top of the graph represent the ratio pS:pT:pY obtained in each enrichment; left axis represents the number of phosphopeptides and phosphoproteins in each experiment (from three technical replicates); right axis expresses the enrichment selectivity of each experiment. LC-MS/MS analysis was performed on a Q Exactive mass spectrometer.

To evaluate the sensitivity of the protocol, the phosphopeptide enrichment was assessed on increasing quantities of MM protein digests (12.5–200 μg) taken from a pool of eight MM tumor lysates (characteristics of the tissue samples used in the pool are described in S1 Table). A pooled sample was generated to avoid a bias towards the characteristics of a single tissue characteristic and to obtain data that could be extrapolated to different MM tissues. As shown in Fig 2, the method was sufficiently sensitive to identify more than 1,000 phosphopeptides from 12.5 μg of input material. As expected, the number of identifications increased when more input material was used. The samples displayed linearity up to 100 μg of loaded peptides. When higher quantities were injected onto the cartridges, however, linearity was compromised suggesting that the overall capacity was affected (S1 Fig). The same trend has been previously observed with cell lines, thus reflecting the credibility of our results and the robustness of the methodology [19]. For all peptide amounts analyzed, the enrichment selectivity was >90%, however, this value may be compromised when lower quantities of material are analyzed. An assumption such as this can be explained partly by the fact that a poor MS signal-to-noise ratio markedly affects phosphopeptide identification [19]. The enrichment from 100 μg of the MM peptides resulted in the best data. Over 5,226 unique phosphopeptides and 5,524 phosphosites class I were identified from a single 120 min LC-MS/MS analysis. Excluding slight differences in phosphopeptide ratios, for all peptide quantities loaded onto the Fe(III)-NTA cartridges the enrichment of phosphorylated serine (pS), threonine (pT) and tyrosine (pY) followed the expected pattern whereby pS>pT>>pY. The same observations were made with respect to the number of phosphopeptides that were identified containing multiple phosphorylation sites (S2 Table).

In summary, the factors discussed above suggest that the optimal performance of the cartridges is in the range of 12.5–100 μg of input material without any compromise in the overall enrichment efficiency. Additionally, quantities less than 100 μg still produced high-quality results and are ideal for cases where very limited quantity of starting material are available.

### Analysis of the MM phosphoproteome from individual tumor samples

**Fig 3:**
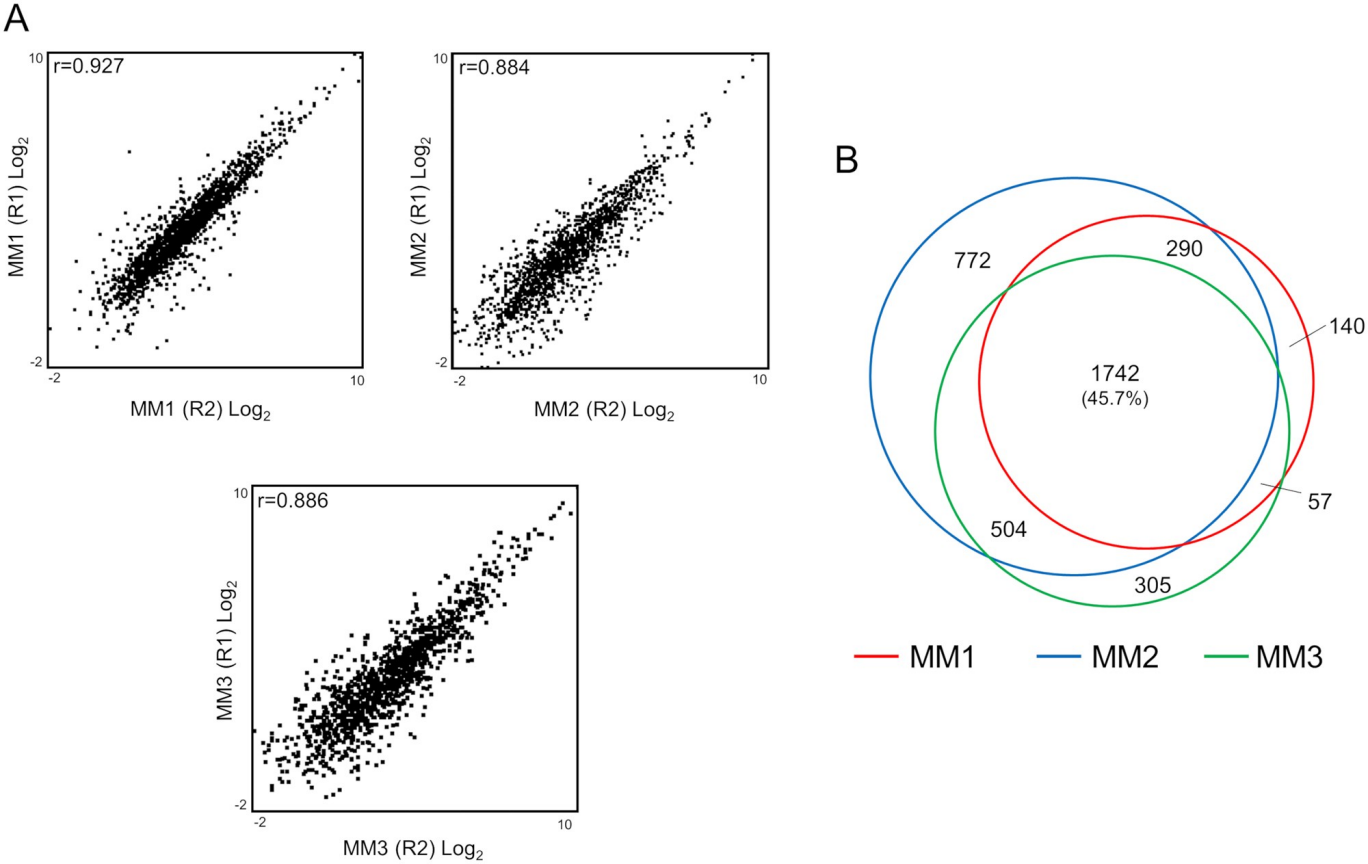
Correlation and overlapping between the three MM samples analyzed. (A) Pearson correlation between independently-enriched samples for the three different MM tissues. (B) Overlap between the phosphoproteomes of the three MM samples.

On the basis of the results obtained from the sensitivity analysis, phosphopeptide enrichment from three different MM tissues was performed (two independent experiments in each tissue). The description of the MM tumors used here are provided in S3 Table. From 60 μg of input material, the data was outstanding with more than 5,600 phosphopeptides and 9,000 phosphosites identified per sample. The three enrichments reflected similar trends in selectivity, pS:pT:pY ratio, and proportion of phosphosite class I and phosphosite multiplicity (Table 1). The reproducibility of the enrichments was assessed by correlation analyses of the quantitated phosphopeptide intensities across independent replicates. Good correlations (Pearson correlation, r>0.88) and a uniform distribution was observed for each comparison, indicating a high reproducibility between experiments (Fig 3A and S3 Fig). These data reflect the consistency of the enrichment across the samples. In this sense, the physical characteristics of the MM tissues appeared to not have any significant influence on the entire sample preparation process.

**Table 1:** Phosphopeptide enrichment for each of the analyzed MM samples. Data represented here was obtained from merging two independent biological replicates.

Description	Sample		
	MM1	MM2	MM3
Total peptides	5,816	12,512	8,614
Total phosphopeptides	5,638	12,201	8,242
% Phosphopeptides	96.9	97.5	95.6
Total proteins (groups)	2,268	3,599	2,831
Phosphosite multiplicity	Mono: 5,076 Di: 521 Tri: 39 Tetra: 2	Mono: 10,370 Di: 1,561 Tri: 245 Tetra: 25	Mono: 7,100 Di: 1,016 Tri: 116 Tetra: 10
Ratio phosphosites (pS: pT: pY)	38: 7: 1	63: 9: 1	58: 9 1
Total phosphosites	9,082	17,669	12,164
% Phosphosites Class I	71	71	75
Starting material (μg)	60		
Instrument	Q Exactive	Q Exactive HF-X	

**Fig 4:**
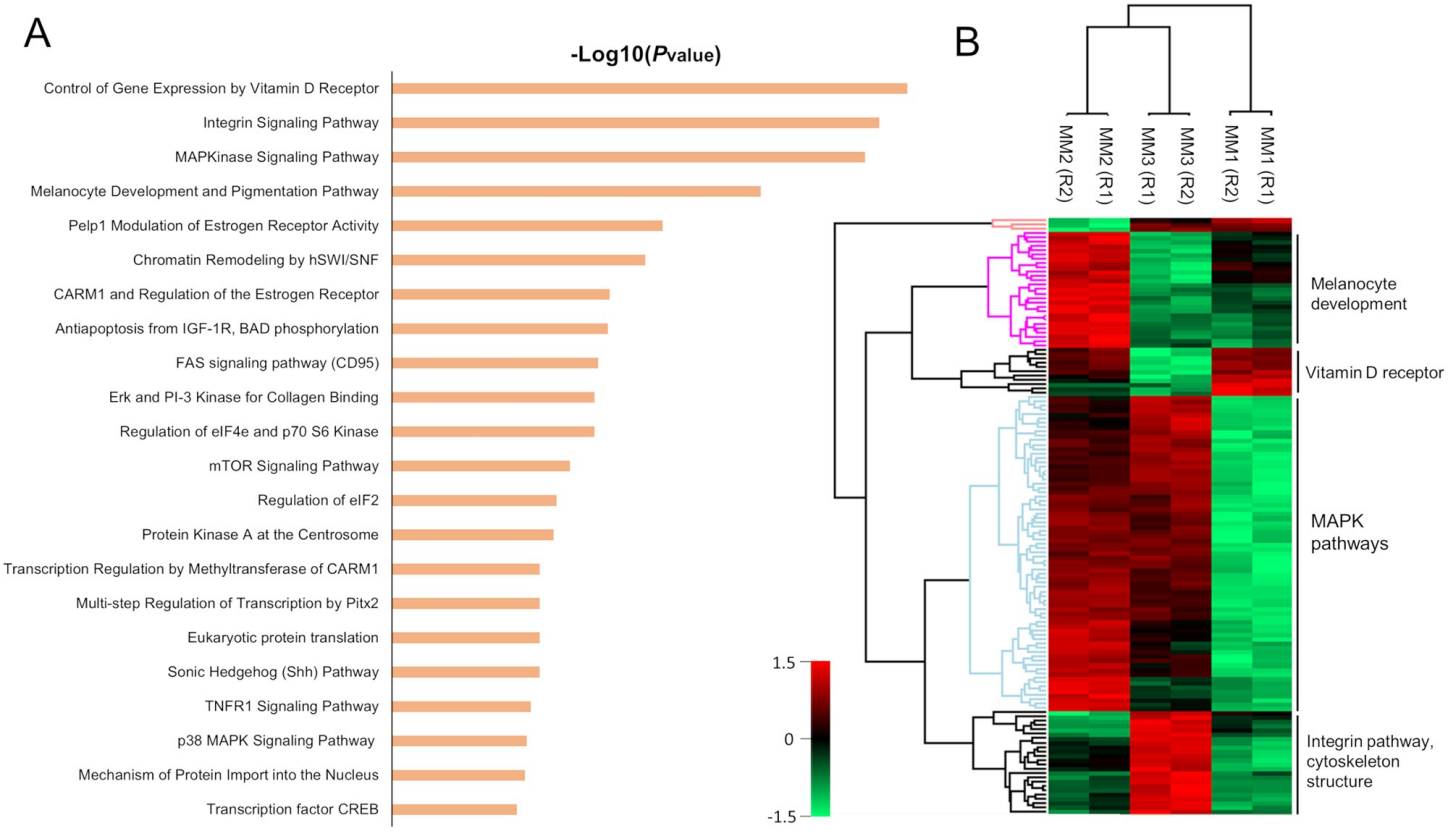
Enriched pathways and clustering of common proteins in MM samples. (A) Enriched pathways related to common proteins identified in MM1, MM2 and MM3. (B) Heat map clustering of proteins with high significance amongst the MM samples obtained from ANOVA analysis (permutation-based FDR >0.01).

In general, the overlap of the identified phosphopeptides between replicates and between patients was higher than 73% and 45%, respectively (S2 Fig and Fig 3B). In principle, the variation amongst the samples could be explained by the differences within the tissues and the performance of the mass spectrometer that was used. Despite these variations, the high overlap between the three samples enabled both pathway enrichment analysis and a quantitative comparison of the phosphoproteomes to detect protein clusters with biological significance. As summarized in Fig 4A, the main pathways that were commonly-enriched for the identified phosphoproteins in the three samples were related to aspects of the melanoma phenotype. Namely, mitogen-activated protein kinases (MAPK) signaling pathways, control of gene expression by vitamin D receptor, integrin signaling, melanocyte development and pigmentation *etc*.

MAPK pathways are essential in the development of MM because of the involvement in cell proliferation and evasion of apoptosis [3]. Proteins that belong to the classical MAPK pathway, such as v-Raf murine sarcoma viral oncogene product (BRAF) and the downstream partners extracellular signal-regulated kinases 1 and 2 (ERK1 and ERK2) were all observed in our data set. These proteins are extremely relevant because of the role played in the activation of several transcription factors that leads to cell proliferation and cell development [22]. Considering that BRAF is mutated in 50–60% of melanomas [23], it is not unreasonable that major drug development has focused on targeting these protein variants. Hence, vemurafenib, trametinib [24], dabrafenib [25], and ipilimumab [26] have been approved or considered as potential cancer therapies against MM. Moreover, MAPK variants were also observed in our samples. In the p38 MAPK cascade, the phosphorylation of AKT, PTP, MEKK1, MKK6, 7, 3 and 4 and p38 are relevant in regulating inflammation, apoptosis and cell cycle steps [22]. In particular, it is known that pAKT acts as an oncogenic signal that promotes tumor progression [27]. In addition, this cascade allows the activation of p53, which is one of the best known cancer suppressors [28]. This protein remains controversial in MM as it is widely present in MM patients but only with very rare mutations. In this sense, the protein remain silent in 90% of MM patients but reactivation combined with BRAF^V600E^ inhibition leads to apoptosis and suppression of melanoma growth [28].

Other enriched MAPK-related pathways were related to proliferation, *e*.*g*., ERK5 and some scaffold proteins (HGK, HPK1 and MEKK1) that are responsible for activating the Wnt pathway [3]. Synergy between MAPK and Wnt leads to the formation and development of melanoma [3]. In addition, non-canonical Wnt proteins induce the release of exosomes from tumor cells containing pro-angiogenic and immunosuppressive factors [29].

Together with MAPK, another pathway that acts synergistically in the development of MM is the melanocyte development and pigmentation pathway. Key proteins such as proto-oncogene receptor tyrosine kinase KIT were observed. KIT is involved in migration and survival of tumor cells, but in higher metastatic states expression is lost [30]. Other phosphoproteins that belong to this cascade were the melanogenesis associated transcriptor factor (MITF), apoptosis regulator BCL2 and cAMP responsive element binding protein 1 (CREB1) [3].

The integrin β pathway was also identified in our data set. The expression of integrin β1 facilitates metastases (migration and proliferation) in lymph nodes [31]. Several downstream proteins activated by phosphorylated integrin β1 such as tensin, zyxin, talin, vinculin, SRC proto-oncogene and paxillin (all involved in F-actin dynamics during migration and cell division) were also identified [32]. Activation of caveolin by interaction with collagen also activates the classical MAPK pathway by the phosphorylation of RAS/RAF. In this case, SRC acts as a redundant protein recruiting actin stress fibers and phosphorylating ERK1/ERK2 [22,33,34].

ANOVA analysis of the label-free quantitated phosphoproteins that were common to the patient samples reflected interesting results (Fig 4B). The main pathways that were previously mentioned appeared as key clusters with differential expression in the three samples, thus demonstrating the relevance of these proteins in MM phenotypes. The differences in expression could potentially be explained by several factors related to the characteristic of each patient (sex, age, percentage of tumor in the tissues, treatment, survival, *etc*.). Unfortunately, the number of samples used here is not sufficient to perform a truly robust statistical analysis to correlate proteins or clusters with clinical insights. Methodologically speaking however, the results are valuable because identification/quantitation of significant differences in phosphoproteomic profile of MM samples regardless of the clinical or physical characteristics of the samples was feasible.

### Deep coverage of the MM phosphoproteome via basic reversed-phase fractionation

**Fig 5:**
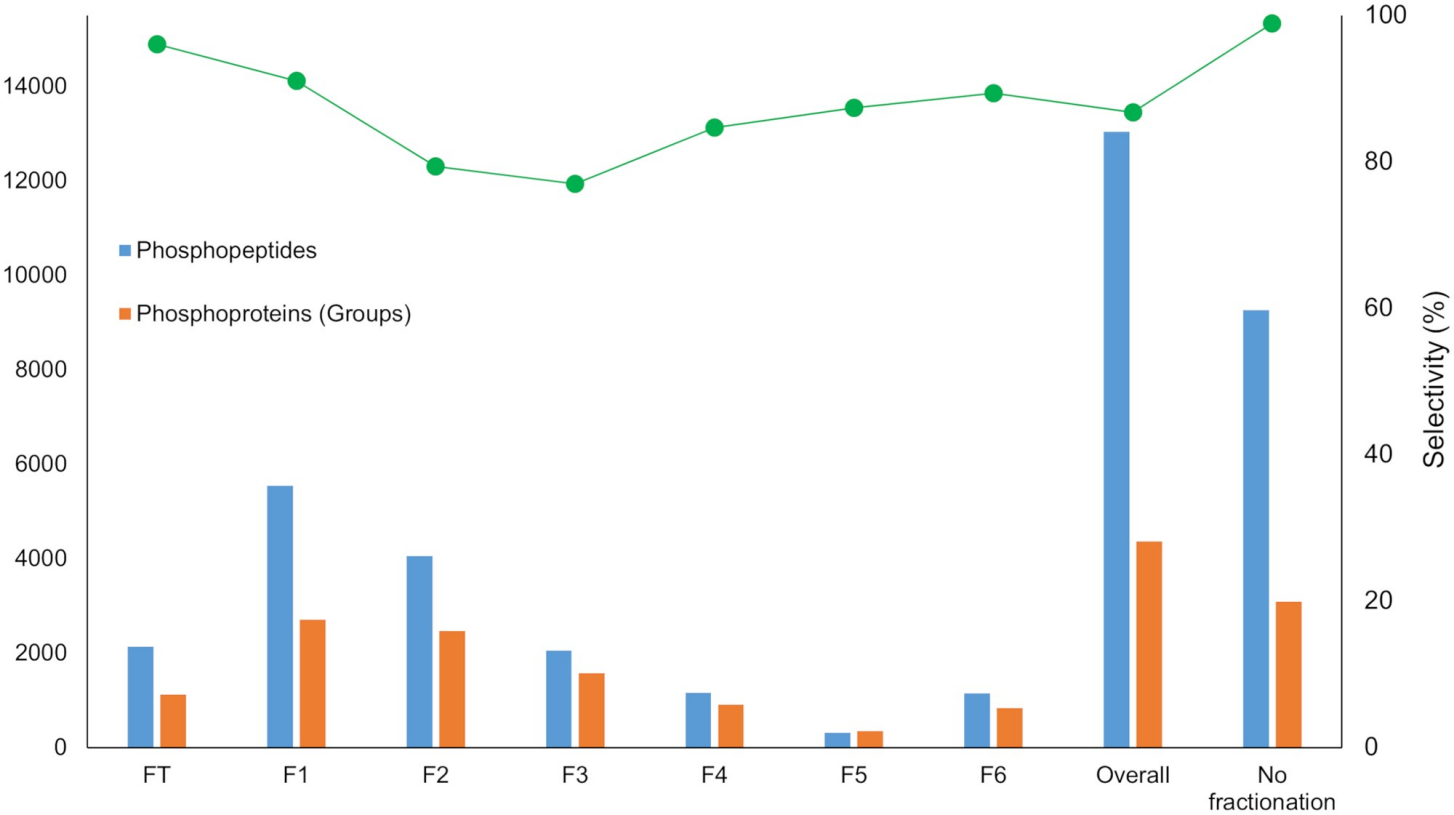
Profile of the phosphopeptide enrichment from the six fractions and the FT obtained from HpH fractionation on the Bravo AssayMap platform for sample MM2. LC-MS/MS analysis was performed on a Q Exactive HF-X mass spectrometer. Results are compared with the overall analysis and the sample without fractionation. Data corresponds to a single replicate.

To increase the depth of the analysis with respect to the number of phosphopeptide identifications, high pH reversed-phase peptide pre-fractionation of the MM2 sample was also performed on the AssayMAP Bravo platform. Application of an optimized step elution method (see Materials and methods) resulted in the collection of seven fractions that included the flow-through. Using the Fe(III)-NTA cartridges, phosphopeptides were enriched from each fraction. Fig 5 and S4 Table show the number of phosphopeptides that were identified from each individual fraction, the overall analysis and in the sample without fractionation. From 60 ug of starting material, 13,036 phosphopeptides and 4,359 phosphoproteins were identified. Compared to the unfractionated sample (8,723 ± 600 phosphopeptides per replicate), this represents an increase of 49% in the identification of phosphopeptides and phosphoproteins.

**Fig 6:**
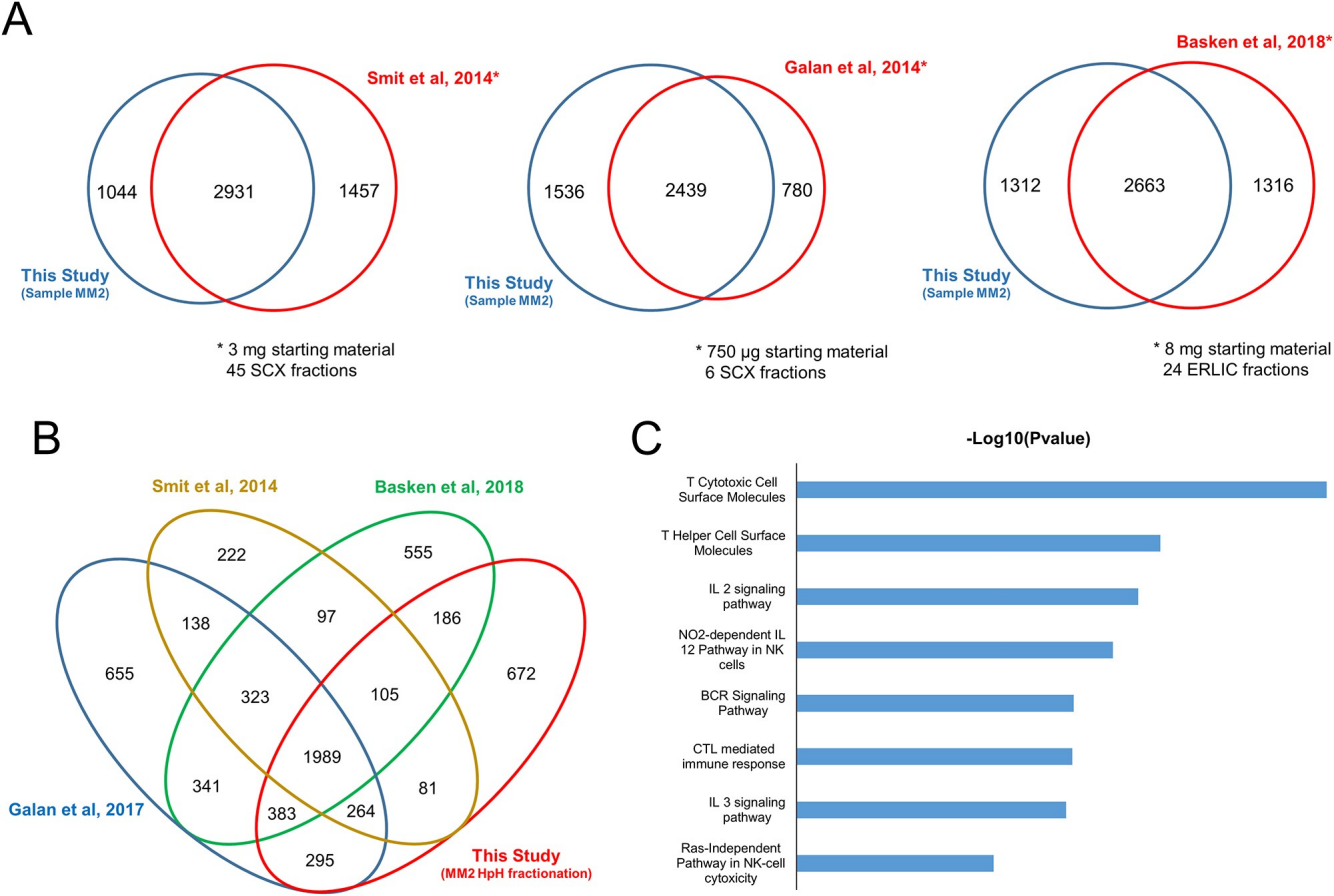
Comparison with previously reported phosphoproteome data sets. (A) Venn diagram comparison of three different reported phosphoproteome data sets from melanoma-derived cell lines with data obtained from HpH-fractionated sample MM2 (single biological replicate). Each comparison is accompanied by the quantity of starting material and the reported fractionation method. (B) Overall comparison between the phosphoproteomic data sets and HpH-fractionated MM2. (C) Enriched pathways related to proteins exclusively identified in our study.

This data set was compared with other available high-throughput phosphoproteomic analyses involving MM cell lines [9–11]. The numbers obtained here are relatively close to those obtained in previous studies (Fig 6A and S5 Table), with approximately 90% of the proteins identified in this experiment observed in prior studies utilizing various approaches and methods. This result is beyond expectations because 12.5- to 100-fold less starting material was used. Similarly, the fractionation method used here was not as extensive as has been shown in earlier studies. Interestingly, 672 proteins were exclusively detected in our data set (Fig 6A and 6B). These proteins are involved in pathways and biological processes related to immunological responses from T cells, B cells and NK cells plus interleukin (IL) pathways (Fig 6C). As the phosphoproteomic analysis in our study was performed on a tissue sample, it was possible to enrich for pathways closely-related to lymph node functions. By using tissues rather than cultured cells, processes that cannot be fully explored with cells were immediately apparent. This is simply because cell culture do not truly reflect the *in vivo* environment of the disease.

As lymph nodes are the major sites of B and T lymphocytes and represent the most frequent early metastatic sites in MM, it is not unreasonable to assume that the antitumor immune response is relevant for the reduction of metastases [35]. Several molecules involved in the progression of antitumor response were identified, *i*.*e*., CD8, CD2, CD11a, CD18, CD45, protein tyrosine phosphatase receptor type C (in T cytotoxic and T helper cells), JAK and STAT proteins, CD3, CXCR3, LCK proto-oncogene, MEK and IL-2R (IL-2 response and NO-dependent IL-12 activation of NK cells). These immune cells play an important role in combatting MM as these assist in establishing a first line anti-tumor immune response in lymph nodes [36]. Immunological treatment of MM that targets some of these proteins is under investigation. Interleukins such as IL-2 or IL-15 are candidates for therapeutic applications based on cytokine action, but severe side effects are associated with systemic cytokine treatment [37]. This ‘exclusive’ data set provides complementary information regarding the biology of MM and enables the exploration of alternative/potential targets in immunological treatment of MM.

Despite a lower number of identified phosphopeptides in the unfractionated sample, sufficient information was still obtained to provide satisfactory biological relevance and context. The comparison with other studies revealed that a significant number of proteins (468 proteins, S4 Fig) were still exclusively identified by this approach. In this context, processing samples without fractionation is a reasonable option to study the phosphoproteome of MM. In particular, such an option would be more practical when many samples, *e*.*g*., from a large patient cohort, would need to be simultaneously processed and analyzed.

## Conclusion

Clinical proteomic studies require large sample cohorts to discover disease-related mechanisms and biomarkers of clinical significance. Thus, it is essential that the field progresses towards a high degree of sensitivity plus robustness in sample preparation. Realistically, this can only be achieved with automated technologies. Hence, it is our belief that instruments such as the Bravo AssayMap platform could play a key role in the analysis of large cohorts of challenging biological samples such as MM. An automated phosphorylation enrichment protocol was assessed. Here, Fe(III)-NTA IMAC cartridges were used in the Bravo AssayMap system and combined with high-performance MS instruments. This method proved to be efficient, sensitive and reproducible in tissue samples obtained from patients with MM. The protocol was robust and enabled the identification of thousands of phosphorylated peptides from a limited quantity of starting material. The MM tissue-based phosphoproteome covered essential features of MM biology and was comparable to reports based on MM-derived cell lines. More importantly, additional MM-related pathways were revealed that can only be detected in human tissue. Overall, our study represents a significant step towards a deeper understanding of malignant melanoma and the opportunity to integrate clinical and phosphoproteomic data in biomarker discovery.

## Supporting information

S1 Fig Linearity of the Fe(III)-NTA IMAC-enriched and quantitated phosphopeptides on the Bravo AsssayMap platform. Only phosphopeptides identified in all replicates were considered for this analysis. All values were normalized with respect to the initial 200 μg protein quantity (intensity = 1). The enrichment displayed quantitative linear performance up to 100 μg of input material.
(TIF)

S2 Fig Venn diagram comparison of identified phosphoproteins in each replicate for the three patient tissues that were analyzed. (TIF)

S3 Fig Normalized protein intensities for each biological replicate of the three MM tumors (only including common proteins identified in all replicates). (TIF)

S4 Fig Venn diagram comparison of melanoma-derived cell line data obtained from major published phosphoproteomic data sets with the data obtained from sample MM2 without fractionation. (TIF)

S1 Table Overall description of MM tissues used in the pool to perform sensitivity analysis of phosphopeptide enrichment. Some patient clinical information is also provided.
(DOCX)

S2 Table Detailed results of the sensitivity analysis of Fe(III)-NTA IMAC phosphoenrichment performed on the Bravo AssayMap platform. (DOCX)

S3 Table Overall description of MM tissues used in the phosphopeptide enrichment of individual samples. Some patient clinical information is also provided.
(DOCX)

S4 Table Detailed description of the phosphoproteome obtained from Fe(III)-NTA IMAC phosphoenrichment on the Bravo AssayMap platform combined with HpH fractionation. (DOCX)

S5 Table Overall comparison of other available high-throughput phosphoproteomic analyses involving MM cell lines with this study. (DOCX)
