# Correction: Automated phosphopeptide enrichment from minute quantities of frozen malignant melanoma tissue

**DOI:** 10.1371/journal.pone.0210234

**Published:** 2018-12-31

**Authors:** Jimmy Rodriguez Murillo, Magdalena Kuras, Melinda Rezeli, Tasso Miliotis, Lazaro Betancourt, Gyorgy Marko-Varga

There is an error in the fourth author’s name in the author byline and citation. The correct byline is:

Jimmy Rodriguez Murillo, Magdalena Kuras, Melinda Rezeli, Tasso Miliotis, Lazaro Betancourt, Gyorgy Marko-Varga

The correct citation is:

Murillo JR, Kuras M, Rezeli M, Miliotis T, Betancourt L, Marko-Varga G (2018) Automated phosphopeptide enrichment from minute quantities of frozen malignant melanoma tissue. PLoS ONE 13(12): e0208562. https://doi.org/10.1371/journal.pone.0208562

## References

[1] Murillo JR, Kuras M, Rezeli M, Milliotis T, Betancourt L, Marko-Varga G (2018) Automated phosphopeptide enrichment from minute quantities of frozen malignant melanoma tissue. PLoS ONE 13(12): e0208562 10.1371/journal.pone.0208562 30532160 PMC6287822

