# Supplementary figures and images for: Automated phosphopeptide enrichment from minute quantities of frozen malignant melanoma tissue
Source: PLoS One. 2018 Dec 10;13(12):e0208562. doi: 10.1371/journal.pone.0208562 (PMC6287822; doi:10.1371/journal.pone.0208562)

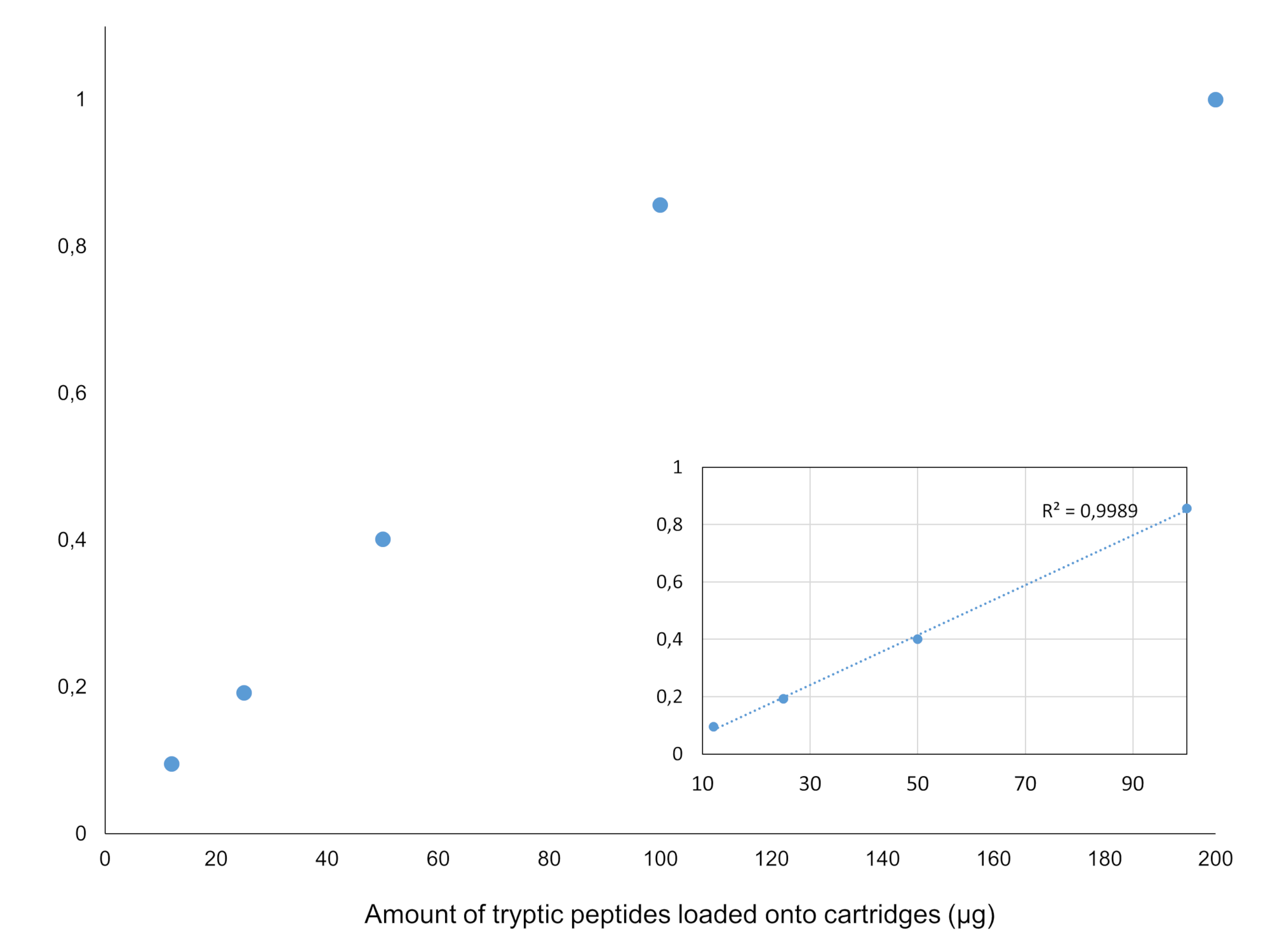

Supplement: S1 Fig — Only phosphopeptides identified in all replicates were considered for this analysis. All values were normalized with respect to the initial 200 μg protein quantity (intensity = 1). The enrichment displayed quantitative linear performance up to 100 μg of input material. (TIF) [file pone.0208562.s001.tif]

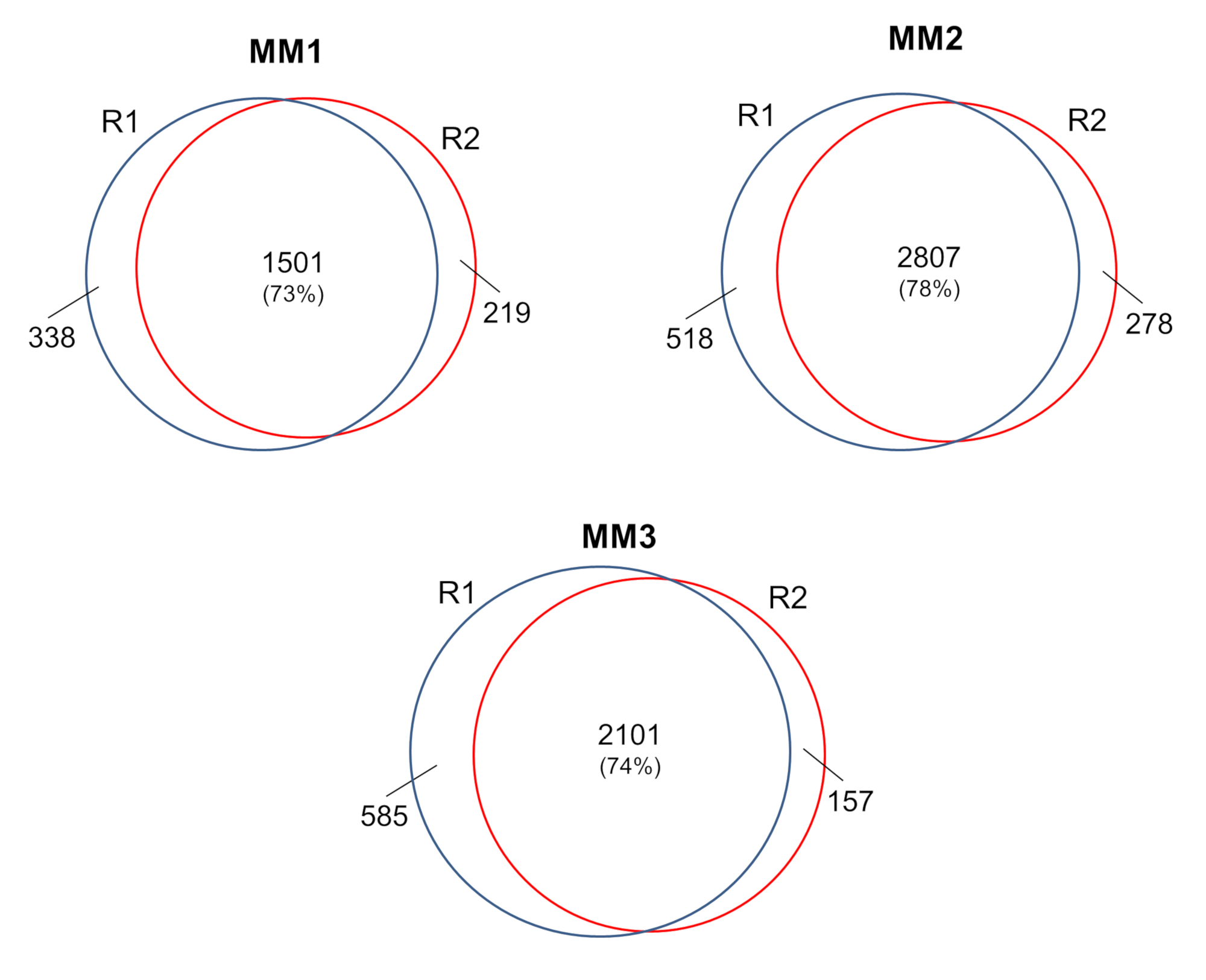

Supplement: S2 Fig — (TIF) [file pone.0208562.s002.tif]

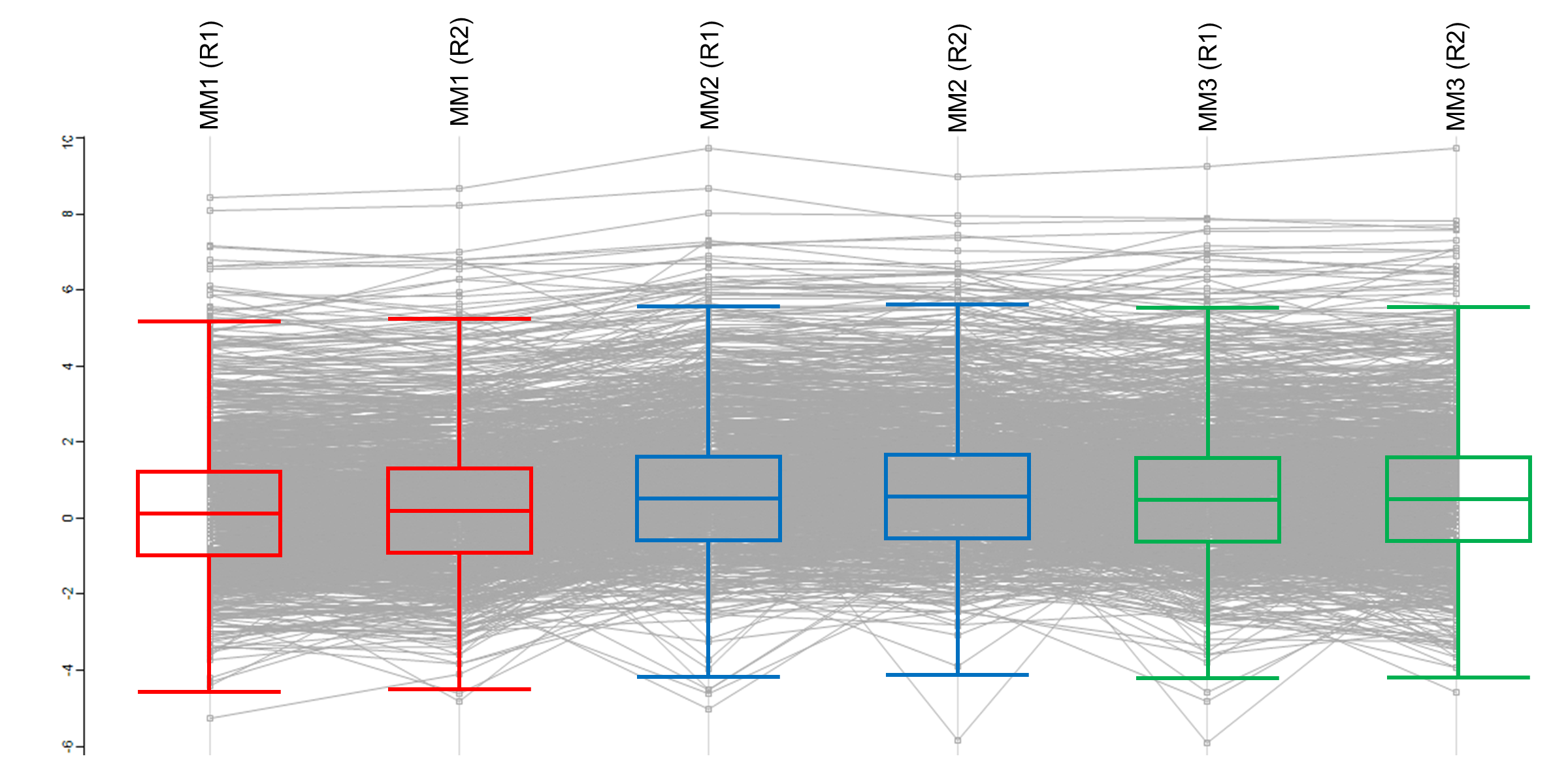

Supplement: S3 Fig — (TIF) [file pone.0208562.s003.tif]

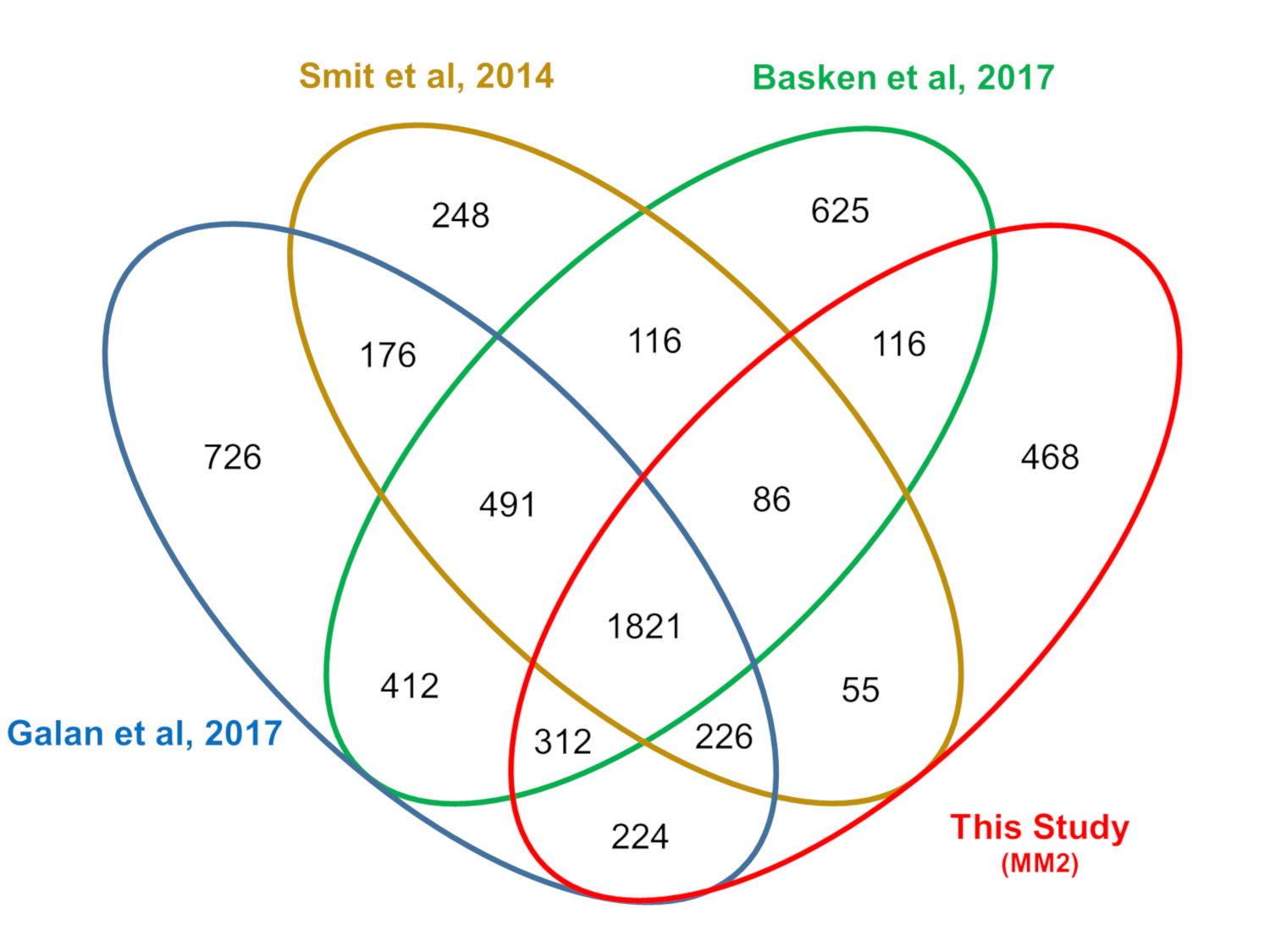

Supplement: S4 Fig — (TIF) [file pone.0208562.s004.tif]
