# Supplementary material for: Automated phosphopeptide enrichment from minute quantities of frozen malignant melanoma tissue
Source: PLoS One. 2018 Dec 10;13(12):e0208562. doi: 10.1371/journal.pone.0208562 (PMC6287822; doi:10.1371/journal.pone.0208562)
Supplement: S1 Table — Some patient clinical information is also provided. (DOCX) [file pone.0208562.s005.docx]

**S1 Table.**

| **Sample** | **Tissue** | | | | **Patient Information** | | | |
| --- | --- | --- | --- | --- | --- | --- | --- | --- |
|  | **Tumor (%)** | **Adjacent lymph node area (%)** | **Necrosis (%)** | **Connective tissue content (%)** | **Age diagnosis** | **Sex** | **Stage** | **Survival** |
| 1 | 96 | 0 | 0 | 4 | 71 | Male | 4 | N |
| 2 | 55 | 0 | 10 | 35 | 55 | Male | 3 | Y |
| 3 | 97 | 0 | 0 | 3 | 55 | Male | 3 | Y |
| 4 | 94 | 0 | 0 | 6 | 53 | Male | 3 | N |
| 5 | 87 | 7 | 0 | 6 | 65 | Male | 4 | N |
| 6 | 86 | 7 | 0 | 7 | 65 | Male | 3 | N |
| 7 | 63 | 27 | 2 | 8 | 51 | Male | 3 | Y |
| 8 | 96 | 0 | 0 | 4 | 71 | Male | 4 | N |
