# Supplementary material for: Automated phosphopeptide enrichment from minute quantities of frozen malignant melanoma tissue
Source: PLoS One. 2018 Dec 10;13(12):e0208562. doi: 10.1371/journal.pone.0208562 (PMC6287822; doi:10.1371/journal.pone.0208562)
Supplement: S2 Table — (DOCX) [file pone.0208562.s006.docx]

**S2 Table.**

| Description/Loaded material | **12.5 µg** | **25 µg** | **50 µg** | **100 µg** | **200 µg** |
| --- | --- | --- | --- | --- | --- |
| Total peptides | 1,226 | 2,770 | 4,097 | 5,305 | 5,081 |
| Total phosphopeptides | 1,139 | 2,631 | 3,988 | 5,226 | 5,005 |
| % Phosphopeptides | 93 | 95 | 97.3 | 98.5 | 98.5 |
| Total proteins (groups) | 716 | 1,359 | 1,778 | 2,061 | 1,947 |
| Phosphosite multiplicity | # mono: 1,074  # di: 61  # tri: 4 | # mono: 2,475  # di: 148  # tri: 8 | # mono: 3,630  # di: 339  # tri: 18 | # mono: 4,503  # di: 636  # tri: 81  # tetra: 6 | # mono: 4,053  # di: 787  # tri: 143  # tetra: 22 |
| Ratio phosphosites (pS : pT : pY) | 48 : 10 : 1 | 44 : 9 : 1 | 38 : 7 : 1 | 46 : 7 : 1 | 44 : 7 : 1 |
| #phosphosites | 1,749 | 4,035 | 6,147 | 7,892 | 7,862 |
| Instrument | Q Exactive | | | | |
