# Supplementary material for: Automated phosphopeptide enrichment from minute quantities of frozen malignant melanoma tissue
Source: PLoS One. 2018 Dec 10;13(12):e0208562. doi: 10.1371/journal.pone.0208562 (PMC6287822; doi:10.1371/journal.pone.0208562)
Supplement: S4 Table — (DOCX) [file pone.0208562.s008.docx]

**S4 Table.**

| Total peptides | 15,030 |
| --- | --- |
| Total phosphopeptides | 13,036 |
| % Phosphopeptides | 87 |
| Total proteins (groups) | 4,359 |
| Phosphosite multiplicity | # mono: 11,520  # di: 1,363  # tri: 145  # tetra: 8 |
| Ratio phosphosites (pS : pT : pY) | 41 : 7 : 1 |
| Total Phosphosites | 20,703 |
| % Phosphosites Class I | 73 |
| Starting material (µg) | 60 |
| Instrument | Q Exactive HF-X |
