# Supplementary material for: Automated phosphopeptide enrichment from minute quantities of frozen malignant melanoma tissue
Source: PLoS One. 2018 Dec 10;13(12):e0208562. doi: 10.1371/journal.pone.0208562 (PMC6287822; doi:10.1371/journal.pone.0208562)
Supplement: S5 Table — (DOCX) [file pone.0208562.s009.docx]

**S5 Table.**

| **Description/Study** | **Galan et al., 2014** | **Smit et al., 2014** | **Basken et al., 2018** | **This Study** |
| --- | --- | --- | --- | --- |
| **Sample type** | A375 cells and HEK293 cells | BRAF^V600E^ melanoma cells 04.01, 04.07, 93.03 and 00.08; A375 and HEK293 cells | WM239A metastatic melanoma cells | Human lymph node tissue |
| **Starting amount** | 750 µg | 3 mg | 8 mg | 60 µg |
| **Fractionation method** | SCX | SCX | ERLIC | High pH reversed-phase |
| **Number of fractions** | 6 | 45 | 24 | 7 |
| **Phosphopeptide enrichment method** | TiO_2_ | Ti^4+^-IMAC | TiO_2_ | Fe(III)-NTA-IMAC |
| **Number of biological replicates** | 2 | 3 | 3 | 1 |
| **Quantification method** | SILAC | Label-free | SILAC | N/A |
| **Mass spectrometer** | LTQ-Orbitrap Elite | LTQ-Orbitrap Elite/Velos | Orbitrap Fusion, LTQ-Orbitrap Velos | Q-Exactive HF-X |
| **LC gradient** | 100 min | 180 min | 100 min | 120 min |
